# Neonatal Seizures: Impact on Neurodevelopmental Outcomes

**DOI:** 10.3389/fped.2015.00101

**Published:** 2015-11-23

**Authors:** Seok Kyu Kang, Shilpa D. Kadam

**Affiliations:** ^1^Neuroscience Laboratory, Hugo Moser Research Institute at Kennedy Krieger, Baltimore, MD, USA; ^2^Department of Neurology, Johns Hopkins University School of Medicine, Baltimore, MD, USA

**Keywords:** neonatal seizures, hypoxic–ischemic encephalopathy, neonatal brain injury, co-morbidities

## Abstract

Neonatal period is the most vulnerable time for the occurrence of seizures, and neonatal seizures often pose a clinical challenge both for their acute management and frequency of associated long-term co-morbidities. Etiologies of neonatal seizures are known to play a primary role in the anti-epileptic drug responsiveness and the long-term sequelae. Recent studies have suggested that burden of acute recurrent seizures in neonates may also impact chronic outcomes independent of the etiology. However, not many studies, either clinical or pre-clinical, have addressed the long-term outcomes of neonatal seizures in an etiology-specific manner. In this review, we briefly review the available clinical and pre-clinical research for long-term outcomes following neonatal seizures. As the most frequent cause of acquired neonatal seizures, we focus on the studies evaluating long-term effects of HIE-seizures with the goal to evaluate (1) what parameters evaluated during acute stages of neonatal seizures can reliably be used to predict long-term outcomes? and (2) what available clinical and pre-clinical data are available help determine importance of etiology vs. seizure burdens in long-term sequelae.

## Introduction

**Figure 1:**
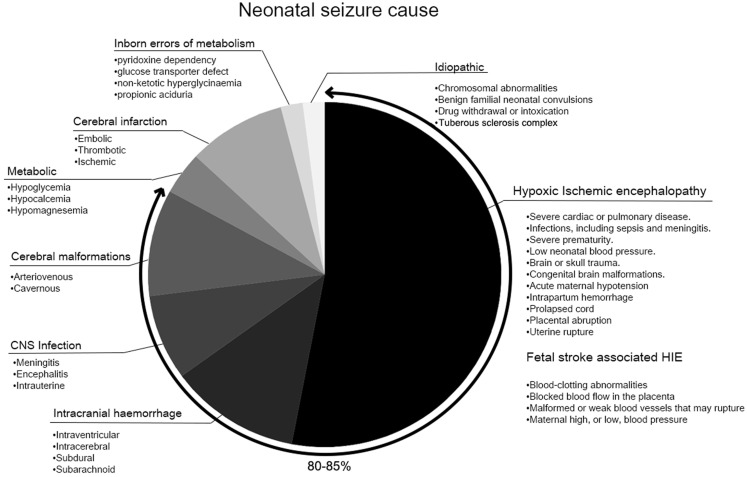
**Neonatal seizure cause**.

The incidence of seizures, 1.5–3/1,000 live births, is highest during the neonatal period (1, 2). Neonatal seizures remain a clinical challenge due to ambiguous presentations and, therefore, sometimes the failure of immediate detection. The lack of evidence-based management protocols, and poor outcomes add to that challenge (3). Most neonatal seizures are symptomatic rather than idiopathic (2) (Figure 1), and 80–85% are predominantly accounted for by hypoxic–ischemic encephalopathy (HIE), hemorrhage, metabolic disturbances, and infections (4, 5). Several mechanisms are known to play a role in seizure initiation in the immature brain.

The immature brain has a higher seizure susceptibility due to multiple developmentally regulated features (1). One of which is the now established excitatory and trophic effect of the GABAergic system during cortical development (6, 7). Phenobarbital (PB) remains the first-line anti-epileptic drug (AED) for neonatal seizures (5, 8), however, with an efficacy of less than 50% (9). Consideration of other AEDs is (10) based on the underlying cause and characteristics of the seizures in neonates. Among many, the use of levetiracetam has increasingly become viable, as studies have reported its safety and efficacy on neonatal seizures associated with HIE and other etiologies (11). Increasing evidence suggests that neonatal seizures are associated with adverse neurodevelopmental outcomes, including epilepsy, cerebral palsy, developmental delay, and psychomotor deficits (12–14). However, whether neonatal seizures can independently impact long-term neurologic outcomes, or are a marker of the severity of underlying pathology, remains a topic of active debate (15, 16).

The lack of evidence-based treatments for neonatal seizures stems both from poor estimation of acute seizure burdens in the absence of continuous EEGs and the refractory nature of neonatal seizures. Without this evidence, the ability to effectively study and predict long-term neurodevelopmental effects of seizures remains a challenge. Clinical and pre-clinical studies on the long-term effects of seizures in neonates are needed to provide conclusive insights on (1) which acutely determined parameters predict the long-term sequelae of neonatal seizures, (2) how aggressively should the acute seizures be treated, and (3) are the current pro-active treatments like hypothermia and repeated doses of AEDs neuroprotective in the long-run?

## Etiology

### Acquired

#### Hypoxic–Ischemic Encephalopathy

Hypoxic–ischemic encephalopathy, reported in 1–2/1,000 live births, is the most prevalent underlying pathology for acquired neonatal seizures (17). HIE-seizures in neonates accompany high seizure burdens with frequent status epilepticus and electrographic seizures (18). HIE-seizures in neonates are known for their resistance to first-line AEDs like PB (19). The alternative treatment options for refractory seizures, such as levetiracetam and midazolam, have shown variable effects (20, 21).

Therapeutic hypothermia (TH) has become a standard practice for treating neonates with HIE, based on the evidence from pre-clinical and clinical studies that documented reduced brain injury in HIE-neonates that underwent TH (22–26). Clinical studies have documented that TH significantly reduced mortality and short-term morbidity, and improved AED efficacy in neonates with HIE (27–30). However, a recent study has reported no significant difference in survival or functional outcome by TH in neonates hospitalized for cardiac arrest (31). The long-term benefits of TH and its effect on chronic outcomes as related to neonatal seizures are awaiting further evaluation.

Pre-clinical modeling allows a thorough evaluation of both acute seizure burdens and the efficacy of treatment protocols with *in vivo* and *in vitro* experiments. Efficacies of AEDs are known to be model specific (32–34) and, therefore, caution must be exercised when making interpretations for translational purposes. Hypoxia is an important component of HIE, and its effect in a developing brain has been studied in a model of neonatal hypoxia (35). Global hypoxia (3–4% O_2_) in P10–12 rats induced acute seizure burden that was mild and age dependent, with no reported brain injury. A long-term study in this model further reported an increased seizure susceptibility to flurothyl-induced seizures but no significant association with neurobehavioral consequences (36). More recent study on this model reported an emergence of spontaneous seizures at juvenile period and significant prevalence of epilepsy at P180 evaluated by EEG (37). Ischemia represents another important cause of HIE. A newer model of neonatal ischemia-alone was characterized in P7, P10, and P12 mice. In contrast to the hypoxia model, ischemia-alone resulted in a status-like seizure burden and PB-resistance associated with neuronal injury (38). Another well-studied model for HIE is the combination of hypoxia and ischemia (HI; Rice–Vannucci model), which has widely been used to study neonatal HIE (39). In P7 rats, HI-induced seizures continued up to 48 h, with significantly decreased background EEG power (40). Lastly, chemoconvulsants have also been used to recapitulate the high-seizure load seen in neonatal HIE (41, 42). Seizures in brain slices, induced by kainic acid or Mg^2+^, displayed high seizure severity with status-like seizure activity and PB-resistance (42, 43). *In vivo* studies of chemoconvulsants, pilocarpine (44) and pentylenetetrazol (45), were also conducted in neonatal rats, but the seizure severity was not quantitated in either study. The characteristics of the seizures studied in these models differ by severity, response to AEDs, and the resultant neuronal injury (33). The long-term co-morbidities were evaluated in only a subset of these studies (Table 1), which is a drawback of some of the models being used and that needs further investigation.

**Table 1:** **Pre-clinical studies of neonatal seizures and their long-term parameters**.

Study	Model	Species	Age of insult	Chronic ages evaluated	Acute EEG seizure	Long-term EEG seizure	Injury	Long-term comorbidities
Stafstrom (6)	Kainic acid	Rat	PND 5, 10, 20, and 30	3 months	N.E	Evaluated	Evaluated CA3 cell loss in P20 and 30	N.E
Jensen et al. (36)	Perinatal hypoxia	Rat	PND 5, 10, and 60	1–2 months	N.E	N.E	N.E	Water maze, open field, handling tests, susceptibility to flurothyl
Lee et al. (46)	Tetanus-toxin	Rat	PND 9–11	Up to 6 months	N.E	N.E	Evaluated No injury	Chronic EEG abnormality
Huang et al. (47)	Flurothyl	Rat	PND 0–9	3 months	50 seizures	N.E	N.E	Increased seizure susceptibility to flurothyl, impaired memory, change in HC morphology
Santos et al. (48)	Pilocarpine	Rat	PND 7–9	3 months	N.E	N.E	Evaluated No injury	Reduced exploratory skills CA1 hyperexcitability
Xiu-Yu et al. (44)	Pilocarpine	Rat	PND 1, 4, and 7	P49	N.E	N.E	Evaluated No injury	Altered neurogenesis
Kadam et al. (49)	Perinatal HI	Rat	PND 7	6 months	N.E	N.E	Evaluated Cortical lesions	Mossy fiber sprouting Cortical dysgenesis
Kadam et al. (49)	Perinatal HI	Mouse	PND12	P33–39	N.E	N.E	Evaluated Hemi: 34% HC: 61%	Rotarod, T-maze alteration, open field, cylinder test
Kadam et al. (49)	Perinatal HI	Rat	PND 7	2–12months	N.E	Evaluated	Evaluated Hemi: 30–78%	N.E
Rakhade et al. (37)	Perinatal hypoxia	Rat	PND 10	3–6 months	N.E	Evaluated	Evaluated No injury	Significant prevalence of epilepsy Increased mossy fiber sprouting in CA3 HC
Lugo et al. (50)	Flurothyl	Mouse	PND7–11	P40	N.E	N.E	N.E	Deficits in HC-dependent memory and social behavior
Kang et al. (38, 51)	Ischemia	Mouse	PND 7, 10, and 12	N.E	Evaluated	N.E	Evaluated P18	N.E
Bernard et al. (52)	Kainic acid	Rat	PND 7	P6090	N.E	N.E	N.E	Abnormal social interaction and restricted interests
Peng et al. (53)	Perinatal HI	Mouse	PND 7	11–12 months	N.E	Evaluated	Evaluated 11–12 month Hemi: 44–69%	N.E

*N.E, not evaluated; HC, hippocampus*.

#### Acquired Non-HIE

##### CNS Infection (Neonatal Bacteremia and Meningitis)

Neonatal meningitis, occurring in every 0.25–1/1,000 live births, is a condition in which seizures are often detected (54) and long-term sequelae, such as hydrocephaly, brain edema, and subdural effusion, follow. *Escherichia coli* and group B *Streptococcus* are typical pathogens for bacterial meningitis and ~25% of neonates with meningitis suffer neurologic complications (55). Administration of dexamethasone, a steroid medication as an adjunct is included in current standard therapy, with minimal side effects reported clinically. (56).However, the data for seizure burdens, AEDs given, and evidence of injury are not readily available to allow evaluation of long-term neurologic outcomes for current management protocols. Additionally, dexamethasone has been shown to increase neuronal injury following asphyxia in preterm fetal sheep, despite later onset and shorter duration of acute seizures (57).

CNS inflammation is known to exacerbate seizure activity and the associated neuronal injury. The condition of prenatal intrauterine infection has been studied in a model of bacterial endotoxin lipopolysaccharide (LPS)-induced inflammation in perinatal rodents. Perinatal LPS exposure was reported to increase seizure susceptibility to chemoconvulsants in rats as adults (58). Similarly, it was also shown to have pro-convulsive and epileptogenic action in a rapid kindling model of neonatal seizures (59). In a long-term, *in utero* inflammation induced by a single-dose injection of LPS in CD1 pregnant mice resulted in behavioral abnormalities, chronic brain inflammation, neuronal loss, and impaired sleep structures in rodents (60, 61).

##### Hemorrhage/Trauma

Intracranial hemorrhage occurs in 3.8/10,000 live births and represents ~15% of seizures reported in the neonatal period (62). Infants with intracranial hemorrhage are at high risk for seizures, regardless of the etiology of hemorrhage. Parenchymal injury was independently predictive of acute seizures, and severity of acute seizures predicted later seizures (63).

Currently, no pre-clinical neonatal models are available to examine the association between intracranial hemorrhage and its acute seizure burdens and the long-term outcomes.

##### Metabolic Disorders

Neonatal seizures and epileptic encephalopathy, although rare, are associated with various inborn errors of metabolism (64) which include hypoglycemia and hypocalcemia. However, there is a lack of clinical or pre-clinical studies that provide insights about the acute quantifiable parameters associated with or co-morbidities caused by neonatal seizures due to metabolic causes.

##### Cortical Malformations

Developmental malformations of the brain are a cause of neonatal seizures with later development of refractory epilepsy (65). The majority of patients suffer developmental disabilities and epileptic seizures following cortical malformations at early ages (66). No clinical reports are available to evaluate acute parameters and long-term outcomes of seizures related to cortical malformations, possibly due to the variability of seizure locus and the seizure onset. Yet, epilepsy surgery has been reported to improve long-term seizure outcome in patients with focal cortical dysplasia (67).

The pathophysiology of cortical malformation has been characterized in neonatal freeze-lesion model in which microgyrus were surgically induced and hyperexcitability were observed (68). Long-term comorbidities associated with this model were reported in studies that evaluated long-term epileptogenesis (55, 69).

#### Neonatal Seizures – Genetic

##### Benign Familial Neonatal Seizures

Benign familial neonatal seizures constitute a small subset of neonatal seizures, often resulting in relatively favorable outcomes with spontaneous remission and normal psychomotor development (70, 71). The prevalent mutations identified include KCNQ2/3 and SCN2A, critical genes for ion channel subunits (72). Recent study on KCNQ2 mutation-positive families reported variable seizure onset and burden, although a higher seizure load at neonatal period suggested higher chance of developing seizures later in life (73). KCNQ2 mutations were also associated with epileptic encephalopathy, and KCNQ2 encephalopathy often manifests refractory seizures, cortical abnormalities, and severe neurodevelopmental delay (74–76).

Kcnq2 knock-out mice were lethal at perinatal stage, but conditional deletions of KCNQ2 channels induced neuronal hyperexcitability in cortical and CA1 pyramidal neurons with abnormal electrocorticogram activity and early death (77). Kcnq2 deficiency resulted in a significant downregulation of KCNQ3/5 protein expression levels, highlighting the critical function of KCNQ2 in maintaining normal neuronal excitability.

##### Tuberous Sclerosis Complex

Tuberous sclerosis complex (TSC), caused by mutations in TSC1 or TSC2, affects 1 in 6,000 live births (78). During early infancy, the majority of TSC patients manifested seizures that were refractory and recurring after remission (79). Brain MRIs of TSC patients have revealed focal cortical dysplasia (80), which often suggested worse neurodevelopmental outcomes such as epilepsy, cognitive impairment, and autism spectrum disorders (81, 82). TSC patients develop autism phenotypes, including cognitive deficits and anxiety (83). Longitudinal studies documented that the earlier and the more severe seizures predicted worse intellectual development (84, 85), this may be associated with long-term abnormal white matter development (86). Additionally, TSC tubers have been classified as sub-types based on their MRI properties. Type C cortical tubers are more likely to be associated with infantile spasms and epilepsy and associated with a worse phenotype (87). Therefore, both EEG and MRI may be good predictors for long-term prognosis for TSC.

Pre-clinical studies using conditional knock-out models of TSC1 or TSC2 have reported hyperactivation of mTORC1 signaling along with developmental abnormalities and lower seizure threshold (88, 89). However, very few of the rodent models elicit spontaneous seizures and, therefore, their impact on outcomes remains unknown.

### Long-Term Co-Morbidities: Seizure Severity, and Injury

The long-term neurodevelopmental sequelae of neonatal seizures are prevalent (2). Nevertheless, very few clinical studies have evaluated the long-term outcomes of neonatal seizures by acute seizure burden and etiology (15). The severity of etiology, seizure burden, and brain injury are known to significantly affect the chronic outcomes, but distinguishing and understanding the role of each individual parameter on the long-term outcomes without standardized protocols across study centers are not feasible.

The underlying etiology has been determined to be one of the main prognostic factors for long-term sequelae in survivors of neonatal seizures (5, 90, 91). HIE, hemorrhage, CNS infection, and cerebral malformations are known to be associated with adverse outcomes compared to other etiologies of neonatal seizures (90) (Figure 1). Grades of neonatal encephalopathy assessed by encephalopathy scores or Sarnat staging are often used to predict neurodevelopmental outcome (92). The effect of hypothermia on improved AED efficacy was shown to depend on the severity of HIE, effective only in neonates with moderate, but not in severe HIE. (93). However, the standardized methodology for identifying the severity of HIE is not uniform. Additionally, severe HIE tends to associate with higher seizure burdens, as is the case in the study by Srinivasakumar et al. Therefore, it is difficult to conclude that etiology was the sole main factor and seizure burden did not exacerbate the encephalopathy.

Neonatal seizures are a significant risk factor for long-term sequelae, especially in the setting of HIE (17). The recurrent seizures themselves appear to cause additional neurodevelopmental consequences beyond that due to the underlying etiology (94). Prolonged seizures were shown to worsen brain damage in HIE brain (95, 96); indicating seizures themselves may have a harmful effect. HIE associated with status epilepticus frequently results in adverse neurodevelopmental outcomes (97, 98). The severity of clinical seizures comprehensively measured by seizure frequency, onset, EEG abnormalities, and number of AEDs used, was independently associated with the brain injury in HIE-neonates (95, 99). The temporal profile of electrographic seizure burdens in neonatal HIE has also been evaluated (18). Differential outcomes associated with the differential timing of onset of seizures, however, are not clear from these studies. Hence, increasing evidence suggests that neonatal seizures need to be controlled, to lessen the long-term co-morbidities above and beyond those associated with the underlying etiology alone (100, 101). Additionally, seizures in a developing brain can beget seizures (102, 103), and, therefore, it is difficult to delineate the role of the underlying etiology vs. prolonged repetitive seizures under these conditions.

Neonatal seizures, especially those that are PB resistant, significantly correlate to moderate–severe brain injury rather than mild or no injury (104). This study found that, the efficacy of a single dose of 20 mg/kg PB significantly differed by the severity of injury. Seizures were readily controlled in neonates with mild or no injury, whereas only 30% of neonates with moderate–severe injury responded to PB. Similarly, the severity of brain injury dictated the seizure burden recorded by video-EEG (93). The presence of brain injury and status epilepticus were highly predictive of the development of epilepsy later on in life (105). Neonatal MRI has demonstrated its possible clinical use for early identification of preterm babies at risk for later cognitive impairment (106). Similar protocols scanning neonates with seizures will help assess long-term outcomes more reliably.

The risk factors that can be used as parameters for predicting chronic outcomes of neonatal seizures remain unclear. A large cohort study at a tertiary center by Nunes et al. reported that the development of postnatal epilepsy and global developmental delay are common following neonatal seizures (107). For both co-morbidities, low birth weight, abnormal postnatal EEG and neuroimaging were also significant risk factors. Follow-up MRIs at 1 and 2 years of age with no evidence of lesion has been reported (108) to indicate better prognostication compared to those with detectable lesions. In a similar study, evaluating risk factors for the long-term sequelae following neonatal seizures, low Apgar score at 5 min, cesarean section, time of seizure onset, seizure type, and the abnormal background EEG were independently predictive of worse long-term outcome following neonatal seizures (90, 109). In line with this observation, lack of EEG recordings for seizure burden quantitation seems like a critical limitation for the interpretations made by studies where EEG seizure burden was not known (15). The identification and quantification of neonatal seizures are heavily dependent on quantitative EEG (15, 110), which remains the gold standard for determining seizure burdens. Additionally, other parameters such as initial injury severity, acute AED efficacy, and follow-up imaging can help provide important insights to help assess role of seizures in long-term outcomes. The severity of etiology, seizure burden, and brain injury can all affect the long-term outcomes of neonatal seizures. The grading of etiology at acute stages reflects the degree of brain injury and seizures are a significant risk factor for later brain injury as assessed by MRI (104, 111).

### Using Pre-Clinical Models to Determine Long-Term Co-Morbidities Following Neonatal Seizures

In a hypoxia model of neonatal seizures, an increased seizure susceptibility was detected at 2 months post-hypoxia, but no neurobehavioral consequences or neuronal cell death (112) (Table 1). In another study using combined HI, the long-term effects of seizures were monitored with radio-telemetry for up to 12 months after seizure induction in P7 rats (49). This study reported that perinatal HI resulted in brain injury that ranged from 30 to 78% and temporally progressive epilepsy. However, the injury severity did not correlate to the severity of seizure rates of the chronic post-stroke epilepsy. But more importantly, the study showed that if the perinatal HI insult did not result in an infarct injury, no epilepsy was detected in such rats even with 1 year of continuous monitoring. One similar study using neonatal HI model has recently shown that brain injury can develop at later stages, 11 months post HI insult (53). Motor seizures were identified only in animals with cystic infarct, but none in the animals without infarct.

In pre-clinical models using chemoconvulsants (flurothyl and kainic acid), seizures in P7–11 rodents led to impaired social interaction and learning tested at P60 (50, 52), supporting the notion that the early life seizures may be associated with autism spectrum disorder and intellectual disability (113). By contrast, mTOR pathway was shown to be involved in the development of autistic-like behavior and chronic epilepsy in a model of neonatal hypoxia induced at P10 (114).

## Conclusion

Lack of evidence-based or standardized clinical protocols for neonatal seizure management, poor efficacy of currently used AEDs, and dearth of clinical studies looking at long-term comorbidities, specifically by neonatal seizure severity and etiology, remain. Pre-clinical models have become the focus of research for investigating effects of neonatal seizures and novel therapeutics to subdue them efficaciously (51, 115, 116). The need for new pre-clinical models that are translationally viable is a critical need in the field (117). Since neonatal seizures are predominantly sub-clinical, EEG recording of electrographic seizures is crucial for estimating the true seizure burdens. Acute EEG seizure burdens are a good indicator of the severity of HIE. Additionally, evaluation of amplitude EEG (aEEG), with its potential benefit of easier application and interpretation, may enhance clinical management of neonatal seizures and prognosis of the outcomes (118). aEEG, a bedside neurophysiology tool that uses a limited number of channels to record raw EEG signal, is easy to record and interpret, without input from a neurologist. However, the limited sensitivity for seizure detection by aEEG makes conventional EEG the most reliable and globally used diagnostic and quantitative measure for neonatal seizures. Follow-up MRIs are a reliable indicator of the associated long-term brain injury. Diverse underlying etiologies of neonatal seizures may result in different types and severities of seizures, and, therefore, various long-term outcomes. Certain non-HIE related seizures may not result in severe long-term co-morbidities and, hence, etiology plays a critical role. However, lack of long-term data following rigorous acute standardized monitoring and treatment protocols hinders our ability to comprehensively understand these differences. Better pre-clinical modeling of neonatal pathologies that lead to neonatal seizures is already a benchmark set by the NIH (117, 119). As related to this review, the important guidelines highlighted for future pre-clinical studies are (1) whether the model recapitulates clinical comorbidities associated with neonatal seizures and (2) if available, whether certain treatments can prevent or reverse such consequences. The development of treatments to prevent long-term co-morbidities in patients at risk from a neonatal brain insult is a major unmet clinical need (100).

## Author Contributions

SKK and SDK contributed equally to the writing of this review. SDK supervised and made final edits.

## Conflict of Interest Statement

The authors declare that the research was conducted in the absence of any commercial or financial relationships that could be construed as a potential conflict of interest.
